# A randomized, placebo controlled trial of omega-3 fatty acids in the treatment of young children with autism

**DOI:** 10.1186/s13229-015-0010-7

**Published:** 2015-03-21

**Authors:** Deepali Mankad, Annie Dupuis, Sharon Smile, Wendy Roberts, Jessica Brian, Toni Lui, Lisa Genore, Dina Zaghloul, Alana Iaboni, Peggy Margaret A Marcon, Evdokia Anagnostou

**Affiliations:** Bloorview Research Institute, Holland Bloorview Kids Rehabilitation Hospital, 150 Kilgour Road, Toronto, M4G 1R8 ON Canada; Clinical Research Services, Hospital for Sick Children, Dalla Lana School of Public Health, University of Toronto, Toronto, Canada; Holland Bloorview Kids Rehabilitation Hospital, Toronto, Canada; The Hospital for Sick Children, Toronto, Canada; Department of Paediatrics, University of Toronto, Toronto, Canada; Division of Gastroenterology, Hepatology and Nutrition, Hospital for Sick Children, Toronto, Canada

**Keywords:** Omega-3, Autism spectrum disorder, Complementary alternative treatment, Randomized controlled trial

## Abstract

**Background:**

Autism spectrum disorder (ASD) is a neurodevelopmental disorder affecting more than 1% of children. It is characterized by social communication deficits and repetitive behaviors/restricted interests. In the absence of any medications known to improve core symptom domains, parents often use complementary alternative treatments, including omega-3 fatty acid supplements.

**Methods:**

We conducted a 6-month, randomized, placebo controlled trial of omega-3 fatty acid supplements (1.5 g) vs placebo in children 2 to 5 years of age with ASD. Primary outcome measures included the autism composite score of the Pervasive Developmental Disorders Behavioral Inventory (PDDBI) and the externalizing problems score of the Behavior Assessment System for Children (BASC-2). Secondary outcome measures included clinical global improvement (Clinical Global Impression-Improvement (CGI-I)), adaptive function (Vineland Adaptive Behavior Scale (VABS-II)), and language gains (Preschool Language Scale (PLS-4)), as well as safety. Exploratory analysis investigated potential correlations between changes in cytokine profiles and treatment response.

**Results:**

Thirty-eight participants were randomized in a 1:1 fashion. There was no significant difference between groups on the 0- to 24-week change in PDDBI autism composite scores (*p* = 0.5). There was a significant group by week interaction on the BASC-2 externalizing problem score, with participants randomized to the treatment group demonstrating worsening scores (*p* = 0.02). There was no statistically significant week by group effect on either adaptive function (*p* = 0.09) or language (*p* = 0.6). Omega-3s were relatively well tolerated. Changes in cytokines during the study did not significantly correlate with treatment response.

**Conclusions:**

This study does not support high dose supplementation of omega-3 fatty acids in young children with ASD.

**Trial registration:**

Clinicaltrials.gov NCT01248728. Registered 22 November 2010.

**Electronic supplementary material:**

The online version of this article (doi:10.1186/s13229-015-0010-7) contains supplementary material, which is available to authorized users.

## Background

Autism spectrum disorder (ASD) is a neurodevelopmental disorder characterized by social communication deficits and repetitive behaviors/restricted interests [1]. Although prevalence estimates vary, recent estimates suggest that 1 in 68 children are diagnosed with ASD in the US. The disorder is evident in early childhood and children often present with a series of co-occurring symptoms and disorders including externalizing behaviors, anxiety, obsessive compulsive symptoms, seizures, sleep and gastrointestinal complaints, and immune differences, among others [2]. There is a paucity of medications shown to be useful for core symptom domains, and only two medications have received an Food and Drug Administration (FDA) indication for the treatment of associated symptoms in ASD (risperidone and aripiprazole for irritability). Within this context, there is extensive use of alternative and complementary treatments for children with ASD [3], even in the absence of good quality data to support their use.

Omega-3 fatty acids are a special class of poly-unsaturated fatty acids and include alpha-linoleic acid (ALA), docosahexaenoic acid (DHA), and eisosapentaenoic acid (EPA). DHA and EPA are found in fish oils whereas ALA is plant-derived. Although it is possible to convert ALA into DHA and EPA, this process is extremely inefficient and we mostly depend on dietary intake of EPA and DHA; only 5% of ALA is converted to EPA or DHA in humans [4]. There is accumulating data to support that EPA and DHA are important for brain structure and function and have been advocated for the treatment of multiple neurodevelopmental disorders including mood disorders, schizophrenia, ADHD, and ASD. DHA and EPA are orthomolecules; their functional sites are exclusively cell membranes. The fundamental importance of DHA for the brain is beyond dispute. The neurons are continually forming axons and dendritic extensions with accompanying cell membranes. Growing membranes must be relatively fluid, and DHA is the most fluidizing element in cell membranes. Even the synapses are made from membranes preferentially enriched in DHA [5]. During the last trimester of fetal life and the first two years of childhood, the brain undergoes a period of rapid growth. DHA is required for the development of the sensory, perceptual, cognitive, and motor neural systems during the brain growth spurt [6,7]. EPA’s importance for the brain’s development *in utero* is unclear, but colostrum and breast milk do contain EPA [8,9]; it plays a significant role in brain function, and its derivatives are key regulators of immune endocrine and cardiovascular function [10]. Several other mechanisms of action have been hypothesized for, including change in neurotransmitter binding and gene expression, and anti-inflammatory actions [11-13].

A series of studies have raised the question of omega-3 fatty acid deficiency in children with ASD. Specifically, Vancassel *et al*. and Bell *et al*. [14,15] reported lower levels of polyunsaturated fatty acids including omega-3 s in children with autism compared to typically developing healthy controls. Ashwood *et al*. [16] failed to replicate that finding in 2006, although there was some evidence for such a disturbance in children with a history of regression. The reason for this variability is unclear, but it suggests the possibility of altered omega-3 fatty acid levels in at least a subgroup of children with ASD. In addition, omega-3 fatty acids have been shown to have anti-inflammatory effects that may include decreasing pro-inflammatory factors, especially IL6, IL10, and TNF-a, and lowering microglia activation [17] all of which would be of interest in ASD [18].

A series of open label studies have previously suggested potential for therapeutic effects of omega-3 fatty acids on language and maladaptive behaviors in children with ASD [15,19,20]. These were followed by three pilot randomized controlled trials. Amminger *et al*. [21] published a small randomized controlled trial of 1.5 g of fish oil supplementation for 6 weeks in 13 children with autism, ages 5 to 17. The study used the Aberrant Behavior Scale (ABC) as the outcome measure and reported trends in improving hyperactivity, although no changes reached statistical significance. Bent *et al.*, [22] reported on a pilot randomized controlled trial in 27 children, ages 3 to 8 with ASD, treated for 12 weeks. They also found a trend for improvement in hyperactivity. In both studies, no effects were noted across social withdrawal, irritability, inappropriate speech, or stereotypy as measured by the ABC [23]. A Cochrane review in 2011 reported no evidence for prescribing omega-3 fatty acids in children with ASD but noted a trend for improvement in hyperactivity and suggested that early treatment after diagnosis may be the time window most likely to provide therapeutic benefit [24]. As such, they reserved judgment for the treatment of young children until such studies maybe available. Lastly, Bent *et al*. [25] reported on an internet-based randomized controlled trial of omega-3 fatty acids in 5- to 8-year olds with ASD. Consistent with previous studies, a trend towards improvement in hyperactivity was noted, although it did not reach statistical significance.

Supplementation with omega-3s has been generally well tolerated. Reported side effects in the pediatric population include gastrointestinal disturbance, headaches, sleep disturbance, fishy breath, and changes in the texture of the skin [13]. Rare adverse events of omega-3 fatty acids include bruising and bleeding diathesis, as they can produce hypocoagulant effects [26].

There have been several questions related to appropriate dosing. Both DHA and EPA seem to be neurologically active with DHA being more important for brain structure in early life and EPA being important for continuing brain function [6,9]. There has been a wide variety of regimens employed in clinical trials across multiple neurodevelopmental/neuropsychiatric disorders, as described above. In adults, doses as high as 9.6 g per day have been used, as in the case of bipolar disorder [27]. The FDA has supported the use of up to 3 g of omega-3 fatty acids per day in adults without medical follow-up. Health Canada has published a monogram in which they recommend up to 1.5 g of EPA plus DHA for children ages 1 to 8 years (http://webprod.hc-sc.gc.ca/nhpid-bdipsn/monoReq.do?id=88).

Given the conflicting data on the potential therapeutic effects of omega-3 fatty acids in ASD and unanswered questions about the timing of treatment, we proceeded with a pilot randomized controlled trial (RCT) of omega-3 fatty acids in preschool-aged children with ASD.

The primary objective of this study was to assess whether omega-3 fatty acids (NutraSea HP) are effective in improving autism symptom severity and externalizing symptoms in young children with ASD. The secondary objective was to evaluate the effect of omega-3 fatty acids on adaptive functioning and language development and to provide further safety data for the use of omega-3 s in preschool-aged children.

In an exploratory fashion, the study aimed to assess whether changes in cytokine levels or omega-3 fatty acid levels in plasma correlate with treatment response.

## Methods

This was a randomized double-blind, placebo controlled trial of omega-3 fatty acids in the treatment of young children with ASD. This study was approved by the Holland Bloorview Research Ethics Board and sponsored by the Alva foundation. The trial was registered prior to enrolling participants at clinicaltrials.gov (NCT01248728).

### Participants

A total of 38 children (28 males and 10 females), 2 to 5 years of age with ASD were randomized into the study, between December 2010 and December 2013. The target sample size of 40 was determined to be adequate to detect effect sizes of 0.8 or larger at 80% power. Participants were recruited from Holland Bloorview Kids Rehabilitation Hospital clinical autism services and partner service organizations. As per the Helsinki agreement, and institutional policy, informed consent was obtained from the parents or guardians of all our participants.

The diagnosis of ASD was established using the DSM-IV (TR) criteria (diagnosis of Autistic disorder or Asperger syndrome) supported by the Autism Diagnostic Observation Schedule (ADOS) [28] and the Autism Diagnostic Interview - Revised (ADI-R) [29]. Overall cognitive level was estimated by the Mullen Scales of Early Learning-AGS edition (MSEL) [30].

Inclusion criteria included a confirmed diagnosis of ASD, age of 2 to 5 years inclusive, on stable non-pharmacologic (educational, behavioral, dietary or natural health product) treatment during the preceding 3 months prior to screening, and a commitment not to initiate or modify interventions during the period of the study. Children had to have a normal physical exam and laboratory tests at screening and parents had to have adequate English proficiency to complete all study assessments. Exclusion criteria included a history of prematurity (less than 35 weeks of gestation), a primary psychiatric disorder other than ASD, use of psychoactive medications, history of significant neurologic, hematological, endocrine, cardiovascular (including any rhythm disorder), respiratory, renal, hepatic, or gastrointestinal disease, coagulation deficits, or a known genetic syndrome. In addition, participants were excluded if they had a known allergy to either the omega-3 fatty acids or placebo components, or if they could not tolerate venipuncture.

### Outcome measures

There were two domains targeted as primary outcomes in the study: autism symptom severity and externalizing behaviors. The rational for this choice was that studies in school-aged children only showed trends for the most part. Therefore, we hypothesized that if we studied children earlier in development, we may have the opportunity to capture larger effect sizes and also potential developmental effects. As such, we chose a measure of autism symptomatology and a measure of externalizing behaviors, both of which are validated in the age group of interest.

Autism symptom severity was measured by the autism composite score of the Pervasive Developmental Disorder-Behavioral Inventory (PDDBI). This is a 188-item extended parent-rated factor-analyzed scale designed to assess treatment outcome in children ages 2 to 12 with ASD. PDDBI is one of the few outcome measures that is validated down to 24 months. It measures both problem behaviors and appropriate social development skills [31,32]. Administration time is approximately 30 to 45 min for the extended form. Its psychometric properties are good and it is considered to have promise as an outcome measure in clinical trials in ASD [2]. The autism composite score was used as the primary outcome measure in this study and the measure was administered at baseline, week 12, and week 24.

The effect of omega-3 fatty acids on externalizing behaviors was measured using the Behavior Assessment System for Children, Second Edition (BASC-2) [33]. This is an informant rating scale that can be completed by parents or teachers, although only the parent form was used in this study. It provides standardized composite scores on Adaptive Skills, Behavioral Symptoms Index, Externalizing Problems, and Internalizing Problems. The externalizing problems subscale was used as the second primary outcome measure in our study. It has strong reliability and validity and has been proposed to be appropriate for use in clinical trials in ASD [2]. This measure was administered at baseline, week 12, and week 24.

Secondary outcome measures included the Vineland Adaptive Behavior Scales, Second Edition (VABS-II) [34] at baseline, week 12, and week 24; the Preschool Language Scale-4 (PLS-4) [35] at baseline and week 24 to minimize burden to the child; and the Clinical Global Impression-Improvement (CGI-I) scale [36] administered at each visit to assess global improvement. The VABS-II is an informant-based instrument designed to measure adaptive behavior in daily settings for children and adults from birth to 90 years of age. It measures behaviors in four domains, Communication, Daily Living Skills, Socialization, and Motor Skills, and provides an adaptive behavior composite standard score. It has strong psychometric properties for both reliability and validity [34]. The PLS-4 is a standardized language assessment that provides a global assessment of a child’s language functioning abilities receptive and expressive language. It has good psychometric properties and is appropriate for children from birth to 6 years 11 months of age [35], although its ability to detect small changes in language acquisition during intervention is not as well established. The CGI-I scale is a seven-point scale that provides a clinician rating of global improvement (CGI-I). It requires the clinician to assess the degree to which the participant’s illness has improved or worsened relative to a baseline state before the intervention. Change is rated as 1, very much improved; 2, much improved; 3, minimally improved; 4, no change; 5, minimally worse; 6, much worse; or 7, very much worse. The CGI scale is a widely used tool in pharmacological trials and has been shown to be robustly sensitive to change in adults and pediatric populations across a wide range of disorders including ASD [36].

Safety and tolerability were evaluated using the Safety Monitoring Uniform Report Form (SMURF) [37]. This is a semi-structured interview that contains a general inquiry, drug-specific queries, as well as several questions about daily activities (for example, sleep, appetite, energy level, bowel and bladder function). This instrument has been widely used in other ASD-related clinical trials.

In an exploratory fashion, a Clinical Global Impression-Improvement focused on gastrointestinal (CGI-I-GI) function was used, and plasma phospholipid omega-3 levels and a cytokine panel were measured at baseline and week 24.

### Randomization

Participants were randomized to the two arms in a 1:1 fashion by the pharmacy, based on a randomization schedule produced by the study biostatistician. They were stratified into one of four strata based on the PDDBI score obtained at screening (four quartiles). Block randomization using blocks of four was used within each stratum in order to ensure that the distribution of autism severity was similar between the two treatment groups. Both research personnel and participants were ‘blinded’ to medication assignment.

### Medication and dosing schedule

Participants started at 0.75 g of EPA + DHA (1.875 ml once a day) of liquid formulation, either NutraSea HP or placebo. If this was well tolerated, the dose was doubled to 1.5 g (3.5 ml) after 2 weeks, as per Health Canada guidelines for maximum dose for this age group. The NutraSea HP formulation is a naturally derived fish oil that is extracted, isolated, and processed to contain EPA/DHA in the ratio of 3:1. The placebo had the same physical characteristics as the medication but contained refined olive oil and medium chain triglycerides. Both the medication and placebo had a natural lemon flavor. Stability, as well as contamination and microbial testing, was done as per Health Canada approvals, during the course of the study.

### Frequency of visits

Participants were seen every 2 weeks for the first 4 weeks and every 4 weeks after that. A table of visits and assessments is included in Supplementary Materials (Additional file 1: Table S1).

### Blood processing and analysis

Blood was centrifuged at room temperature (approximately 21°C) at 3,000 rpm for 10 min immediately after collection. Then, the plasma was aliquoted into cryogenic vials and stored in a −80°C freezer. At the end of the study, a set of samples were sent to Dr Bruce J Holub at University of Guelph. The lipids were extracted, and the omega-3 fatty acids (EPA and DHA) in the plasma phospholipid were analyzed via high performance capillary GLC.

A second set was sent to Dr Jane Foster at McMaster University. Cytokine levels in plasma were measured with enzyme-linked immunosorbent assay (ELISA) kits using standard protocols provided. The kits used were the Human IFN-gamma Quantikine ELISA Kit, Human TNF-alpha Quantikine HS ELISA kit, and Human IL-6 Quantikine ELISA Kit from R & D systems (Minneapolis, USA) and the Human IL-1 beta ELISA and Human IL-10 ELISA kits from Ray Biotech (Norcross, USA).

### Statistical analysis

We calculated the mean, its standard error, and range by treatment group across the following potential confounders: age, MSEL, ADOS, and ADI-R scores. Because of our small sample, we did not rely on statistical significance to compare the two groups but rather examined the distributions qualitatively to determine whether or not there was any evidence of clinically significant group differences that could impact on our results. We controlled for sex in our models despite similar distributions in our two groups because of the known association between sex and externalizing behaviors. This served to reduce the error term in our model, enhancing our power to detect treatment differences.

For the PDDBI, BASC-2, and VABS-II, the change from baseline to weeks 12 and 24 was analyzed using a mixed model regression analysis, with baseline scores and sex in the model as potential confounders. Each analysis had one within person factor (time), and one primary between-person factor (treatment group). A time × treatment group interaction term was included in the model to allow the estimation of group differences in the change from baseline to week 24. For PLS-4, no week 12 data were collected, so a simple linear regression of change from baseline to week 24 controlling for baseline and sex was used to evaluate the group effect.

For the CGI, participants were classified as responders (CGI ≤2) or non-responders (CGI >2). These groups were then compared using a basic 2 × 2 (group × responder) Fisher’s exact test.

The severity of adverse events (AE) for the most severe adverse event over the study period was obtained for each participant, and its distribution was compared between the two groups using a Fisher’s exact test. Similarly, the probability of association with treatment was obtained for the event with the highest probability of being associated with treatment for each participant and compared between groups. Finally, the number of participants who experienced at least one event within each event type was also compared between the two groups.

Lastly, we calculated the difference between pre-and post-study levels of cytokine and fatty acids to report on their correlation with the pre- and post-study difference in the primary outcomes.

## Results

### Patient characteristics

**Figure 1:**
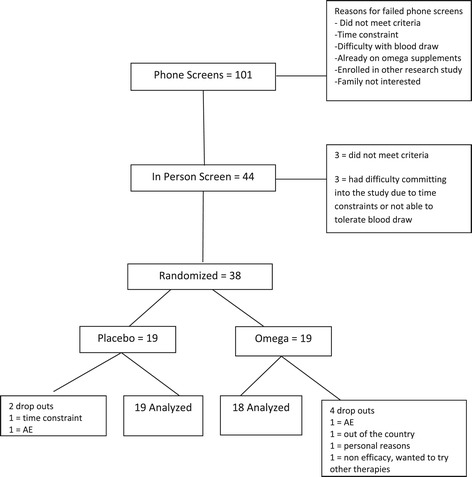
**CONSORT flow chart showing the distribution of participants during the study.**

A total of 101 children were initially screened by telephone, and 44 were invited to be screened in person. Six of these children either did not meet the criteria or had personal reasons not to participate in the study; thus 38 children were randomized in a 1:1 fashion. The mean age of children in the placebo group was 3.5 ± 1.1, and that in the omega-3 group was 3.8 ± 1.0. The majority of enrolled children were male (13/19 in the placebo group, and 15/19 in the omega-3 group). There was no clinically significant difference in baseline characteristics (Table 1, Additional file 2: Table S2). One participant dropped out of treatment in the placebo group due to time constraints at week 12 but participated in an end of study assessment. A second participant dropped out of the placebo treatment due to an adverse event (AE) at week 2. In the medication group, four children did not complete the study. Two withdrew from treatment due to personal reasons, one due to an AE, and one withdrew to explore other treatment options as he experienced no benefit. Two provided end of study assessments and two did not (Figure 1).

**Table 1:** **Baseline characteristics**

	***Placebo (n = 19, 13 males, 6 females)***		***Omega (n = 18, 14 males, 4 females)***	
	**Mean (minimum-maximum)**	**95% CL for mean**	**Mean (minimum-maximum)**	**95% CL for mean**
Age (years)	3.5 (2.0 to 6.0)	3.0 to 4.0	3.9 (2.0 to 4.2)	3.4 to 4.4
ADOS				
Communication (C)	5.5 (3.0 to 9.0)	4.6 to 6.5	5.5 (1.0 to 8.0)	4.6 to 6.5
Social interaction (SI)	9.5 (6.0 to 14.0)	8.4 to 10.6	10.7 (4.0 to 14.0)	9.2 to 12.1
Communication (C) + social interaction (SI)	15.0 (9.0 to 22.0)	13.1 to 16.9	15.2 (5.0 to 22.0)	12.5 to 17.9
ADI-R				
Communication	17.2 (5.0 to 28.0)	14.3 to 20.1	19.4 (9.0 to 29.0)	16.7 to 22.2
Social interaction	12.5 (7.0 to 23.0)	10.5 to 14.5	12.8 (9.0 to 20.0)	11.4 to 14.2
Play	6.2 (2.0 to 10.0)	5.1 to 7.2	5.6 (2.0 to 10.0)	4.7 to 6.4
Stereotyped behavior	4.1 (3.0 to 5.0)	3.7 to 4.5	4.1 (1.0 to 5.0)	3.6 to 4.6
Mullen scales of early learning-AGS				
Visual reception	30.8 (20.0 to 63.0)	23.5 to 38.2	31.7 (19.0 to 65.0)	24.3 to 39.1
Fine motor	28.3 (19.0 to 65.0)	21.9 to 34.6	23.6 (19.0 to 41.0)	20.2 to 27.1
Receptive language	28.8 (19.0 to 56.0)	22.4 to 35.1	27.6 (19.0 to 62.0)	21.5 to 33.7
Expressive language	26.8 (19.0 to 44.0)	22.8 to 30.8	25.7 (19.0 to 45.0)	21.2 to 30.3
Blood omega levels	4.7 (3.4 to 6.2)	4.1 to 5.3	5.5 (3.9 to 8.2)	4.5 to 6.6

### Clinical outcomes

#### Primary outcomes

**Figure 2:**
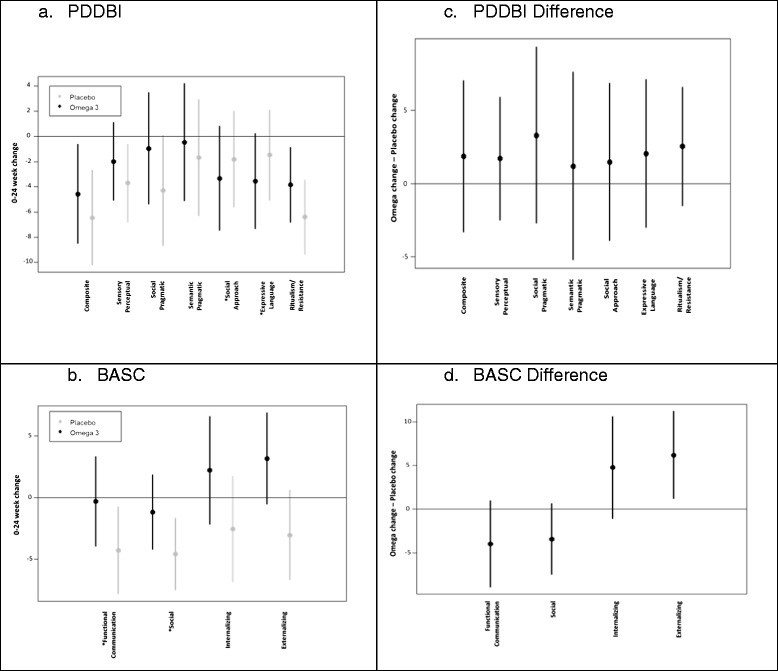
**Omega-3 vs Placebo effects on the PDDBI and BASC-2. (a,b)** Negative change reflects improvement, and **(c,d)** positive values favor placebo. *Items are reversed so that negative change corresponds to an improvement across all PDDBI and BASC items.

There was no significant difference between groups on the 0- to 24-week change in PDDBI autism composite scores (df = 43.4, *t* = −0.82, *p* = 0.5, 95% CI = −3.3 to 7.0) (Table 2, Figure 2). However, there was a significant group by week interaction on the BASC-2 externalizing problem score, with participants randomized to placebo showing a mild improvement (mean change = −3.0 points from baseline), whereas the treatment group demonstrated worsening scores at week 24 (mean change = 3.2 points from baseline), (df = 33.2, *t* = −2.55, *p* = 0.02).

**Table 2:** **Efficacy table**

	**Change from baseline**		**Omega-3 change - placebo change**	***P*** **value**
	***Omega-3***	***Placebo***		
PDDBI (negative change is an improvement)				
*Autism composite*	*−4.5 (−8.5; −0.6)*	*−6.4 (−10.2; −2.7)*	*1.9 (−3.3;7.0)*	*0.5*
Sensory perception	−2.0 (−5.0; 1.1)	−3.7 (−6.7; −0.6)	1.7 (−2.4;5.9)	0.4
Social pragmatic	−0.9 (−5.4; 3.5)	−4.3 (−8.6; 0.1)	3.3 (−2.7;9.3)	0.3
Semantic pragmatic	−0.4 (−5.1; 4.2)	−1.7 (−6.3; 2.9)	1.2 (−5.2;7.6)	0.7
Social approach^a^	−3.3 (−7.4; 0.8)	−1.8 (−5.6; 2.0)	−1.5 (−6.9;3.9)	0.6
Expressive language^a^	−3.5 (−7.3; 0.2)	−1.5 (−5.0; 2.1)	−2.1 (−7.1;3.0)	0.4
Ritualism/Resistance to change	−3.8 (−6.8; −0.9)	−6.4 (−9.3; −3.5)	2.6 (−1.5;6.6)	0.2
BASC (negative change is an improvement)				
Internalizing	2.2 (−2.1; 6.6)	−2.6 (−6.9; 1.7)	4.8 (−1.0;10.6)	0.1
*Externalizing*	*3.2 (−0.5; 6.9)*	*−3.0 (−6.6; 0.6)*	*6.2 (1.3;11.2)*	*0.02* ^a^
Functional communication^a^	−0.3 (−3.9; 3.3)	−4.3 (−7.8; −0.8)	4.0 (−0.9;8.9)	0.1
Social^a^	−1.2 (−4.2; 1.9)	−4.6 (−7.5; −1.7)	3.4 (−0.6;7.4)	0.1
VABS (positive change is an improvement)				
*Adaptive functioning composite*	*2.8 (0.2; 5.3)*	*−0.2 (−2.6; 2.2)*	*3.0 (−0.4;6.4)*	*0.09*
Motor	0.4 (−4.5; 5.4)	−1.9 (−6.6; 2.9)	2.3 (−4.5;9.1)	0.5
Social	−0.3 (−3.1; 2.5)	0.8 (−1.8; 3.4)	−1.1 (−4.8;2.6)	0.6
Communication	1.6 (−1.5; 4.7)	0.9 (−2.0; 3.9)	0.6 (−3.5;4.8)	0.8
Daily living	5.5 (0.9; 10.0)	−0.7 (−5.0; 3.6)	6.2 (0.01;12.3)	0.05
PLS (positive change is an improvement)				
*Total language* *score*	*0.7 (−2.7; 4.1)*	*−0.6 (−3.7; 2.6)*	*1.3 (−3.1;5.7)*	*0.6*

Primary/secondary outcome measures are in italics. All other measures are considered exploratory.

^a^Items are reversed so that negative change corresponds to an improvement across all PDDBI and BASC items.

#### Secondary outcomes

The Adaptive Functioning Composite standard score of the VABS showed no statistically significant week by group effect (df = 51.2, *t* = −1.54, *p* = 0.09). A similar finding was noted on the PLS-4, with no significant week by group interaction observed (df = 31, *t* = −0.60, *p* = 0.6). The CGI-I score was used as a dichotomous variable (1, 2: responder, 3 to 7: non-responder), and no significant difference between groups was observed, *p* = 0.9.

### Safety

There were no serious adverse events (SAEs) reported during the study. Most adverse events reported were mild to moderate in severity and are summarized in Table 3. There was no statistically significant difference between groups in either the severity of the most severe adverse event (*p* = 0.3) or in the distribution of highest probability of association with treatment (*p* = 0.9). There were no statistically significant differences in the prevalence of adverse events across any of the event types (*p* = 0.1 up to *p* > 0.9 across 18 event types). On inspection, there was great variability on both onset and duration of adverse events in both groups and no specific patterns emerged.

**Table 3:** **Adverse events**

**Omega-3**	**Placebo**
Neuropsychiatric disorders	Neuropsychiatric disorders
• Overactivity (3)	• Decreased energy (2)
• Decreased energy (4)	• Social communicative intent loss (1)
• Social communicative intent loss (1)	• Increased emotional lability (3)
• Starring spell (1)	• Increase irritability/anger/aggression (4)
• Headache (1)	
• Stuttering (1)	
• Increase emotional lability (1)	
• Increase irritability/anger/aggression (10)	
Sleep disturbances	Sleep disturbances
• Initial insomnia (6)	• Initial insomnia (4)
• Mid-cycle insomnia (6)	• Mid-cycle insomnia (3)
• Early awakening (1)	• Nightmares (1)
• Non-restorative sleep (1)	
• Nightmares (1)	
Nutritional and gastrointestinal disorders	Nutritional and gastrointestinal disorders
• Increased appetite (1)	• Decreased appetite (3)
• Decreased appetite (7)	• Abdominal pain (4)
• Decreased dietary variety (1)	• Vomiting (3)
• Abdominal pain (6)	• Diarrhea (9)
• Regurgitation (1)	• Constipation (7)
• Vomiting (4)	
• Diarrhea (5)	
• Constipation (7)	
Eyes, ears, nose, teeth, and throat	Eyes, ears, nose, teeth, and throat
• Eye swelling (1)	• Red eyes (1)
• Nearsightedness (1)	• Nose bleed (3)
• Eye surgery (1)	• Tonsillectomy (1)
• Earache (3)	
Allergic	Allergic
• Nasal congestion (2)	• Nasal congestion (1)
• Cough (3)	• Cough (4)
Urogenital disorders	Urogenital disorders
• Day time wetting (1)	• Pain on urination (1)
	• Change in urine colour (1)
Musculoskeletal disorders	Musculoskeletal disorders
• Muscle ache (1)	• Left knee injury (1)
• Right leg internal rotation (2)	
Skin and subcutaneous disorders	Skin and subcutaneous disorders
• Rash (10)	• Rash (9)
• Eczema (2)	• Eczema (4)
• Bruising (4))	• Bruising (2)
• Dry skin (1)	• Dry skin (2)
	• Flaky scalp (1)
Infections	Infections
• Upper respiratory infections (23)	• Upper respiratory infections (24)
• Fever (4)	• Fever (5)
• Ear infection (1)	• Bronchiolitis (3)
• Gastroenteritis (1)	• Ear infection (3)
• Skin infection (1)	• Gastroenteritis (2)
	• Skin infection (1)
	• Hand mouth foot disease (2)
	• Roseola (1)
Serious adverse event: 0	Serious adverse event: 0

Numbers correspond to numbers of distinct incidents.

### Exploratory

There was no statistically significant difference between the treatment and control group (*p* > 0.9) for the CGI-I focused on GI issues.

Omega-3 fatty acid levels were measured at baseline and week 24. Baseline values were similar between the two groups and there was no overlap between the distributions of plasma levels between groups at week 24; therefore, no further analyses were done to relate baseline or change in omega-3 fatty acid level to change in outcome.

In terms of cytokine levels, both negative correlations (an increase in the cytokine level from baseline to week 24 correlated to a decrease in autism-related behaviors) as well as positive correlations were observed; none of which was considered significant when a Bonferroni adjustment was applied (Additional file 3: Table S3).

## Discussion

This is the first randomized controlled trial to our knowledge to study the efficacy and safety of omega-3 fatty acids in children exclusively 5 years or younger, with ASD. It also has the longest study duration (6 months), allowing us to examine effects on functional outcomes. Consistent with previous studies across age ranges, we have no evidence for efficacy of omega-3 fatty acids on core symptom domains. Given that the sample size is small, the possibility always remains that it is responsible for the lack of difference between the groups. For that reason, we explored whether values associated with clinically meaningful change were included in the 95% confidence interval for the omega-3 vs placebo difference at 24 weeks, which was −3.3 to 7. Although there is no published data on what constitutes meaningful change on the autism composite of the PDDBI, extensive discussion with the authors of the instrument suggested that 10 points or 1 standard deviation should be considered clinically meaningful (personal communication, Ira Cohen, 2015). This value is not included in the above confidence interval, which makes it unlikely that the lack of difference between groups is due to sample size limitations. We also found no evidence for efficacy of these compounds on language acquisition, global improvement, or overall adaptive skills over a 6-month period.

However, there was a statistically significant difference in externalizing behaviors, with participants in the placebo group experiencing slight improvements and children in the omega-3 group worsening over the course of the study. This effect is peculiar in the context of previous studies, but is robust. There are several potential explanations for this. All of our participants were 5 years or younger; a group underrepresented in previous studies. As such, it is possible that omega-3 fatty acids at the doses used produced externalizing behaviors in this age group. Secondly, the majority of our participants were minimally verbal. As such, we considered the possibility that potential GI distress, known to be associated with these supplements, may have been under-reported by parents but captured as reports of externalizing behaviors. The link between GI distress and externalizing behaviors in this population has been well established [38]. However, there was no evidence of increase in GI adverse events in the active group even when the group was restricted to non-verbal individuals. Of note though, 8/19 participants in the omega-3 group had GI distress at baseline, and only 1/19 in placebo group had GI distress at baseline. The possibility that preexisting GI distress predisposes to externalizing behaviors cannot be ruled out. Of note, studies in mostly older children have reported trends of improvement in hyperactivity, as discussed in the background. Although this was not a specific goal of this study, we explored the effect of this intervention on hyperactivity as measured by the BASC. There was no statistically significant difference between groups and the trend favored placebo (df = 33.3, *t* = −1.79, *p* = 0.08).

Unlike previous reports [22], there were no consistent or statistically significant correlations between changes in primary outcome measures and changes in cytokines during the study.

The non-overlapping distributions of the difference between week 24 and baseline plasma levels of omega-3 fatty acids would suggest that any dietary intake of omega-3 fatty acids was trivial compared to supplementation and that there was no cross contamination of the active ingredient across groups; that is, participants on placebo did not take omega-3 fatty acid supplements without notifying study staff. As such, our lack of statistically significant differences across domains could not be due to placebo participants having accessed the active ingredient available over the counter.

A limitation of this study stems from the relative paucity of outcome measures focusing on core autism symptomatology for this age group. Although the PDDBI is still relatively a new instrument, it is considered to hold promise in this area [2], but its assay sensitivity is still being established. The small sample size is the primary limitation of the study. Given the number of participants, we could only examine composite scores of various behavioral and adaptive domains. However, there was no consistent trend favoring the active ingredient.

## Conclusions

These data do not support the hypothesis that supplementation of 1.5 g of EPA + DHA in preschoolers with ASD provides any efficacy related to core symptom domains or adaptive function. Our data are not pertinent to nutritional supplementation at levels recommended by various health bodies to assure proper nutrition. There is extensive data supporting adequate omega-3 fatty acid intake across age groups and for young children in particular. Our data, however, would suggest that much higher dosing typically given to children with ASD and advocated as an autism-specific supplementation does not facilitate skill acquisition in this group and may be producing worsening externalizing behaviors.

## Additional files

Additional file 1: Table S1. Schedule of assessments. Documents which instrument utilize participant or parent/guardian as the informant, the rater of the instrument, and the visit schedule of when each instrument is administered. Additional file 2: Table S2. Concomitant treatments. Number of participants receiving each intervention per group, and average exposure to each intervention per group. Additional file 3: Table S3. Cytokine levels. Correlations between change in cytokine levels and 0- to 24-week change in primary outcome measures. A positive correlation corresponds to worsening behavior with increasing cytokine levels.

## Abbreviations

ABC: Abberant Behavior Checklist
ADHD: Attention Deficit Hyperactivity Disorder
ADI-R: Autism Diagnostic Interview, Revised
ADOS-G: Autism Diagnostic Observation Schedule, Generic
AE: adverse event
ALA: alpha-linoleic acid
ASD: autism spectrum disorder
BASC-2: Behavior Assessment System for Children, Second Edition
CGI-I: Clinical Global Impression-Improvement
DHA: docosahexaenoic acid
DSM-IV: Diagnostic and Statistical Manual of Mental Disorders, Fourth Edition
EPA: eisosapentaenoic acid
FDA: Food and Drug Administration
GI: gastrointestinal
MSEL: Mullen Scales of Early Learning
PDDBI: Pervasive Developmental Disorder Behavior Inventory
PLS-4: Preschool Language Scale, Fourth Edition
RCT: randomized control trials
SAE: serious adverse event
SMURF: Safety Monitoring Uniform Rreport Form
VABS-II: Vineland Adaptive Behavior Scales, Second Edition
