# Supplementary material for: A randomized, placebo controlled trial of omega-3 fatty acids in the treatment of young children with autism
Source: Mol Autism. 2015 Mar 21;6:18. doi: 10.1186/s13229-015-0010-7 (PMC4367852; doi:10.1186/s13229-015-0010-7)
Supplement: Additional file 1: Table S1. — Schedule of assessments. Documents which instrument utilize participant or parent/guardian as the informant, the rater of the instrument, and the visit schedule of when each instrument is administered. [file 13229_2015_10_MOESM1_ESM.docx]

Additional file 1: Table S1

**MEASURE INFORMANT RATER VISIT**

**Diagnostic / Intelligence**

DSM-IV Criteria informant (usually parent) TC BL

Autism Diagnostic Interview-R informant Psychologist/ RA BL

Autism Diagnostic Observation Schedule-G participant Psychologist/ RA SC, wk24

Physical/mental health Assessment participant TC SC

Mullen participant Psychologist/RA SC

**Treatment Outcome Measures**

Adverse Event Monitoring (SMURF) informant TC BL, wk2, 4, 8, 12, 16, 20, 24*

Weight, Vital signs participant TC BL, wk 12, 24

CGI Behavioral – Severity informant TC BL

CGI GI – Severity informant TC BL

CGI Behavioral - Improvement informant TC wk 2,4,8,12, 16, 20, 24*

CGI GI – Improvement informant TC wk 12, 24*

PDDBI informant informant BL, wk 12, 24*

BASC informant informant BL, wk,12, 24*

Vineland informant Psychologist/ RA BL, wk 12, 24*

PLS informant Psychologist/ RA BL, wk 24*

*BL: Baseline; *: end visit; RA: research assistant, T.C: treating clinician*
