# Supplementary material for: A randomized, placebo controlled trial of omega-3 fatty acids in the treatment of young children with autism
Source: Mol Autism. 2015 Mar 21;6:18. doi: 10.1186/s13229-015-0010-7 (PMC4367852; doi:10.1186/s13229-015-0010-7)
Supplement: Additional file 2: Table S2. — Concomitant treatments. Number of participants receiving each intervention per group, and average exposure to each intervention per group. [file 13229_2015_10_MOESM2_ESM.docx]

| **Non Pharmacological Concomitant Treatment** | **Omega** | | **Placebo** | |
| --- | --- | --- | --- | --- |
|  | **n** | **Average**  **hours/week** | **n** | **Average hours/week** |
| Intensive Behavior Therapy | 2 | 16 | 2 | 20 |
| Behavior Support | 5 | 7.6 | 6 | 6.5 |
| Social Skills | 1 | 1 | - |  |
| Speech Language Therapy | 12 | 1.4 | 11 | 1.4 |
| Occupational Therapy | 5 | 0.8 | 4 | 0.5 |
| Physiotherapy | 1 | 1 | 1 | 0.5 |
| Therapeutic Learning/Recreational Activity | 2 | 13 | 6 | 8.9 |
| Multivitamins + Minerals | 10 | - | 12 | - |
| Dietary + Probiotics | - | - | 3 | - |

Additional file 2: Table S2: Concomitant Therapies
