# Supplementary material for: A randomized, placebo controlled trial of omega-3 fatty acids in the treatment of young children with autism
Source: Mol Autism. 2015 Mar 21;6:18. doi: 10.1186/s13229-015-0010-7 (PMC4367852; doi:10.1186/s13229-015-0010-7)
Supplement: Additional file 3: Table S3. — Cytokine levels. Correlations between change in cytokine levels and 0- to 24-week change in primary outcome measures. A positive correlation corresponds to worsening behavior with increasing cytokine levels. [file 13229_2015_10_MOESM3_ESM.docx]

Additional file 3: Table S3: Correlations between change in cytokine levels and 0-24 week change in primary outcome measures. A positive correlation corresponds to worsening behaviour with increasing cytokine levels.

|  | **Interferon ᵞ**  R  (P value)* | **IL-6**  R  (P value) | **IL-10**  R  (P value) | **IL-1b**  R  (P value) | **TNF-α**  R  (P value) |
| --- | --- | --- | --- | --- | --- |
| ***PDDBI:***  ***Autism Composite*** | 0.23  (0.2) | - 0.12  (0.5) | -0.07  (0.7) | -0.15  (0.4) | 0.16  (0.3) |
| ***BASC:***  ***Externalizing*** | -0.02  (0.9) | -0.08  (0.6) | 0.35  (0.03) | 0.41  (0.01) | 0.07  (0.66) |

*After Bonferroni correction, p values ≤ 0.005 are considered significant
